# Translational HIV-1 research: from routine diagnostics to new virology insights in Amsterdam, the Netherlands during 1983-2013

**DOI:** 10.1186/1742-4690-10-93

**Published:** 2013-08-28

**Authors:** Antoinette C van der Kuyl, Margreet Bakker, Suzanne Jurriaans, Nicole KT Back, Alexander O Pasternak, Marion Cornelissen, Ben Berkhout

**Affiliations:** 1 Laboratory of Experimental Virology, Academic Medical Center of the University of Amsterdam, Amsterdam, the Netherlands; 2 Department of Medical Microbiology, Laboratory of Clinical Virology, Center for Infection and Immunity Amsterdam (CINIMA), Academic Medical Center of the University of Amsterdam, Meibergdreef 15, 1105 AZ Amsterdam, the Netherlands

**Keywords:** HIV-1, Patient care, Viral load assays, Genotyping, Novel subtypes, Fitness, Superinfection, Seroreversion

## Abstract

An HIV-1 diagnostic laboratory was established in the Academic Medical Center (AMC) of the University of Amsterdam after the discovery of human immunodeficiency virus (HIV) as the cause of the acquired immunodeficiency syndrome (AIDS). The first AIDS patients were diagnosed here in 1981 and since 1983 we have tested the samples of 50992 patients using a variety of assays that greatly improved over the years. We will describe some of the basic results from this diagnostic laboratory and then focus on the spin-off in terms of the development of novel virus assays to detect super-infections and ultra-sensitive assays to measure the intracellular HIV-1 RNA load. We also review several original research findings in the field of HIV-1 virology that stem from initial observations made in the diagnostic unit. This includes the study of genetic defects in the HIV-1 genome and time trends of the replication fitness over 30 years of viral evolution, but also the description of novel HIV-1 variants in difficult-to-diagnose clinical specimen.

## Introduction

The acquired immunodeficiency syndrome (AIDS) was recognized by the Centers for Disease Control (Atlanta, USA) in 1981 [1]. The causative agent, the human immunodeficiency virus (HIV) was isolated first by Barré-Sinoussi *et al* and described as lymphadenopathy-associated virus (LAV) in 1983 [2], and also by Gallo *et al*, who named it human T lymphotropic virus type III, HTLV-III [3]. The complete nucleotide sequence of this novel retrovirus was reported almost two years later [4,5]. The development of antiretroviral compounds in the next decade changed AIDS from a lethal infectious disease to a chronic condition. The first antiretroviral drug, azidothymidine (AZT, zidovudine), a nucleoside analogue targeting the reverse transcriptase (RT) enzyme, was approved by the US Food and Drug Administration in March 1987 for clinical use in AIDS patients [6], and many others rapidly followed. AZT was administered to Dutch AIDS patients since mid-1987 as monotherapy [7,8]. After that, serial monotherapy with novel RT inhibitors was the standard of care, until the development of protease (PR) inhibitors enabled combination therapy to be established [9]. Large scale introduction of HAART (Highly Active AntiRetroviral Therapy), now designated as combination antiretroviral therapy or cART, into the clinical practice in the Netherlands started in June 1996, when the PR inhibitors indinavir, ritonavir, and saquinavir became widely available [10].

From the early days on, it was appreciated that the main feature of AIDS is a steady decline of CD4+ T-cells circulating in peripheral blood, causing a significant change in the CD4+/CD8+ T-cell ratio (see: [11]). CD4+ (and CD8+) T-cell counts were hence used to monitor disease progression. Furthermore, individuals infected with HIV have benefited tremendously from the advances in molecular techniques that became available in the 1980s, which markedly increased the virus assays available for patient care. Plasma viral RNA load (pVL) was found to be another predictor of disease progression [12], and pVL determination became part of routine patient care. After the introduction of antiretroviral drugs, determination of drug-resistance mutations in patient-derived viral genomes by PCR amplification and sequencing of the protease-reverse transcriptase genes (PR/RT genotyping) became yet another standard clinical test [13,14].

This review focuses on the HIV-1 epidemic in the Netherlands and the diagnostics and research performed at the AMC hospital of the University of Amsterdam. However, we should point out that the HIV research field developed rapidly through international efforts that were conducted simultaneously by many centers of clinical care and research. In fact, competition between these international centers has been an important driver of scientific advancement in the area of HIV research.

## Review

### Basic numbers of HIV-1 diagnostics at the AMC hospital

The first three diagnosed Dutch AIDS patients; all hospitalized in the AMC in Amsterdam were described in March 1983 [15-17], two months before the discovery of HIV was published. Two of these patients had been seen by clinicians for AIDS-related complaints since 1981, the third was first seen in 1982 and was probably infected outside the Netherlands [16].

**Figure 1:**
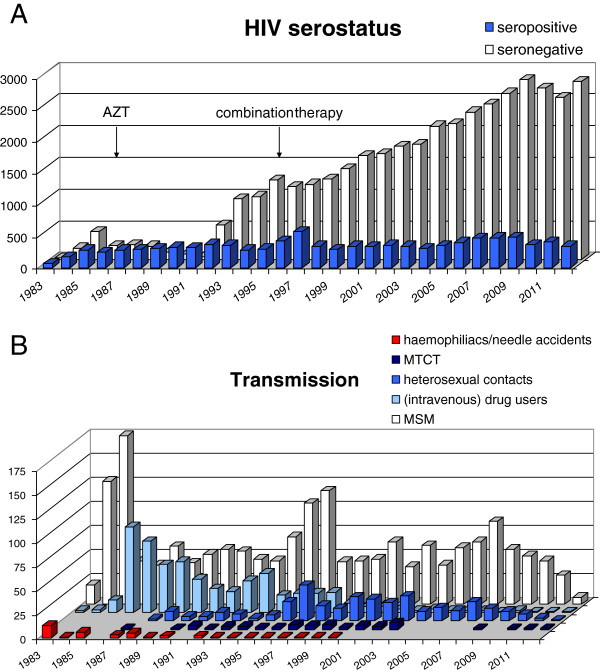
**HIV antibody-tests at the AMC. A**. Numbers of individuals tested for HIV-infection using serology at the AMC (Amsterdam, the Netherlands) from 1983-2012. The introduction of antiretroviral treatment (AZT/zidovudine and cART) is indicated by arrows. Between 1984 and 1992/1993, patient data were mainly recorded in written documents and not always filed later in databases. Therefore, the number of tests in those years is probably an underestimation as not all data could be retrieved. **B**. Numbers of HIV positive patients tested in the AMC in Amsterdam from 1983-2012 according to the most likely route of HIV transmission.

After the discovery of HIV in 1983, serum antibody ELISA and Western blot tests were developed that could diagnose HIV-infected individuals [18]. The total number individuals tested in the AMC from 1984-2012 for antibodies against HIV (both with positive and negative outcome) is plotted in Figure 1A. The number of individuals tested has increased substantially from the 1980s till recent years, but is levelling off in recent years. Figure 1B shows the number of HIV antibody tests performed in relation with the most likely transmission route according to the risk group: men having sex with men (MSM) (homo- or bisexual men), heterosexual contacts (mainly immigrants), (intravenous) drug users (DU), recipients of blood products (haemophiliacs, needle accidents ) and mother-to-child (vertical) transmission (MTCT). In most cases, the risk group is not registered, so that patient numbers shown are much lower than in Figure 1A. Peaks in 1984/1985 and 1998/1999 in numbers of MSM correspond with active recruitment for the Amsterdam Cohort Studies on HIV/AIDS (see below). The AIDS epidemic was initially recognized as being confined to the MSM risk group, patients with haemophilia who received blood products and intravenous drug users, so testing of other risk groups occurred infrequently in the early years of the epidemic. After some years it was realized that other groups such as heterosexuals and children born to infected mothers were also at risk, and the testing policy was expanded accordingly. In 2001, the Municipal Health Service started to offer HIV testing to pregnant women in the Amsterdam area [19]. However, as most refused the test, opt-out testing (patients may elect to decline or defer testing after being notified that the test is routinely performed) was applied in 2003 [19]. Since 2004, all pregnant women in the Netherlands are being tested for HIV using this opt-out policy [20]. Since then, MTCT rates of HIV have decreased in the Netherlands from 5-10 per year to less than 1 per year (Figure 1B). In fact, all children that tested HIV-positive after 2003 were immigrants from non-Western countries.

Testing for HIV-1 is done mainly at outpatient clinics for sexually transmitted infections. These centers include the Municipal Health Service in Amsterdam, where >20.000 HIV tests were performed per year in recent years [21]. Since 2005, HIV rapid tests - based on HIV-antibody detection in saliva (for a review, see [22]) - have been introduced that dramatically increased the number of tests performed [21]. In addition, HIV-tests are requested by general practitioners (GPs), but numbers are much lower. Approximately 11 HIV-tests/10.000 patients per year were GP-requested in 2009 [23]. Self-testing is another way to determine one’s HIV status. A survey under internet users in the Netherlands showed that 71/4416 (1.6%) respondents had ever performed an HIV/AIDS self-test, of which only 9% had used a true home-test [24].

HIV screening assays performed at the AMC are mainly done on patients visiting the outpatient clinics, hospitalized patients, on patients visiting GPs in the neighbourhood and on individuals participating in the Amsterdam Cohort Studies (see below) [25].

HIV-1 infections in the Netherlands are mainly found in MSM. Of the 16,169 HIV-infected patients in clinical care in June 2012, 80% are men of which at least 74% have been infected through homosexual contact according to the HIV Monitoring Foundation Report 2012 [26]. However, HIV-testing, both at the Municipal Health Service and as requested by GPs mostly involves heterosexuals, as high-risk MSM often opt-out [21,23]. It has been estimated that 20-40% of HIV-infected individuals in the Netherlands are unaware of their HIV status [21].

**Figure 2:**
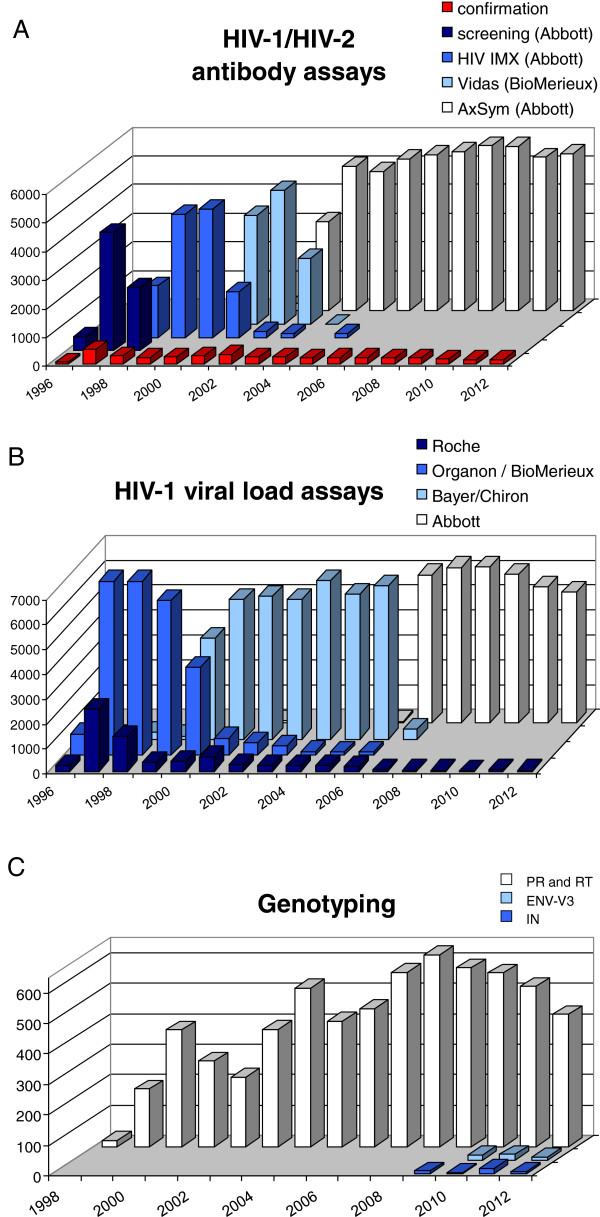
**Numbers of HIV-assays performed at the AMC. A**. Numbers of HIV-1/HIV-2 antibody assays performed in the AMC (Amsterdam, the Netherlands) from 1996-2012. Confirmation assays include Western blots and INNO-LIA (Innogenetics NV, Gent, Belgium). **B**. Numbers of HIV-1 plasma viral load assays performed in the AMC (Amsterdam, the Netherlands) from 1996-2012. **C**. Number of genotyping assays performed in the AMC (Amsterdam, the Netherlands) from 1996-2012. In-house assays were used for IN and ENV-V3, and for PR/RT until a commercial assay became available for the latter genomic region in 2001 (Applied Biosystems ViroSeq HIV-1 Genotyping System).

The numbers of HIV-1/2 screening antibody tests and confirmatory Western blots and other confirmatory assays performed in the AMC since 1996 are plotted in Figure 2A. Antigen tests (HIV-1 p24) became available in 1986 and have been used as a prognostic marker [27,28]. pVL assays became part of the standard patient care around 1996 (Figure 2B). The early peak in the number of assays performed around 1998-1999, reflects the intense testing of the few available patients using a variety of assay formats. Subsequent peaks reflect the increase in the number of patients, usually being tested with a single specific test that screens for both HIV antigen and anti-HIV antibodies.

After the introduction of antiretroviral regimens that contained multiple drugs from 1997 onwards, drug-resistance testing was implemented in patient care to monitor the development of drug-resistance mutations, which is usually due to low compliance. This test also allowed one to investigate the pre-existence of drug-resistance mutations in plasma virus before the start of treatment, so that drug prescription could be adjusted accordingly. Numbers of sequence tests performed are shown in Figure 2C. In recent years, due to the introduction of antiretroviral drugs targeting viral proteins other than PR or RT, sequencing of integrase (IN) and envelope (ENV) gene fragments was performed for a limited number of patients.

Overall, we witnessed a steady increase over time of the number of assays performed, but also a more recent drop. This drop mainly reflects decreasing clinical budgets in this economically challenging period. Consequently, fewer assays are being requested.

### The Amsterdam Cohort Studies

Subsequent analysis of a cohort of homosexual men participating in a hepatitis B virus (HBV) vaccine efficacy trial between 1980 and 1982 suggested that HIV was introduced in the homosexual population in Amsterdam at the end of the 1970s [29]. The Amsterdam Cohort Studies on HIV/AIDS (ACS) were started in October 1984, initially as a two-year prospective study of HIV incidence in MSM [30,31], then as an open, prospective cohort study on HIV. Later, DUs were also included in this cohort, but the HIV-1 incidence in drug users has declined steadily in the Netherlands from 1986 onwards to less than 1.0/100 person-years in 2010 with no new seroconversions after 2009 [32,33]. From 2003-2010, adult patients with primary HIV-1 infection (PHI) from 13 Dutch treatment centres were recruited in a cohort study coordinated by the AMC to analyse the effect of early cART treatment on disease progression [34]. Patient samples from both the ACS and the PHI cohort were subsequently used for in-depth studies of HIV infection characteristics that will be summarized below.

Similar cohorts for the study of HIV infection and AIDS were recruited in other countries, such as the Multicenter AIDS Cohort (MACS), initiated in 1983 at the Johns Hopkins School of Public Health, the University of Pittsburgh School of Public Health, Northwestern University School of Medicine, and the UCLA School of Public Health in the USA [35] and the Swiss HIV Cohort study (SHCS) that was established in 1988 in Switzerland [36]. Also, many cohort studies have been or are being performed in African countries, often in association with western universities.

### Drug-resistance mutations and HIV-1 evolution

After the introduction of the first antiretroviral compounds into the clinic in 1987, it was realized that HIV can rapidly become resistant to single- or double-drug regimens. RT genotyping became part of the standard patient care followed by PR sequencing around 1995. It was implemented for patients failing on therapy or starting treatment, the latter such that optimal regimens could be installed. Also, there was a concern that drug-resistant HIV-1 strains might be transmitted to become stable components of the epidemic. An in-house sequencing assay was used until 2001, when a commercial genotyping assay became available (Applied Biosystems ViroSeq HIV-1 Genotyping System [13,14]).

**Figure 3:**
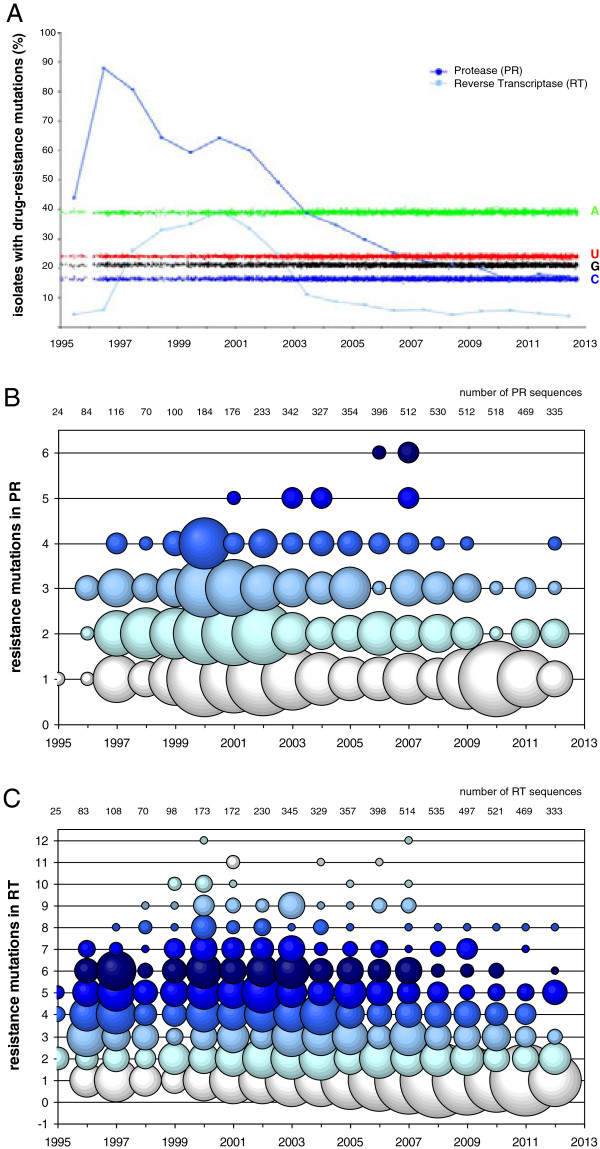
**HIV drug resistance testing in the AMC. A**. Percentage of key drug resistance mutations PR/RT in sequences from 1995-2012. Percentages of A (green), C (blue), U (red) and G (black) nucleotides are also shown. **B**. The number of key drug resistance mutations in PR from 1995-2012. The size of the circles is correlated with the number of sequences in that category. **C**. The number of key drug resistance mutations in RT per sequence from 1995-2012. The size of the circles is correlated with the number of sequences in that category; colours were added for clarity.

In the AMC, a total of 5266 PR/RT sequences from 4226 patients were determined between 1995 and 2012 that could be retrieved from the database. For most of the patients (84.3%) a single PR/RT test was performed, but several patients (10.6%) were tested repeatedly. Due to the early use of monotherapy combined with suboptimal patient adherence, drug-resistance mutations, estimated according to the Stanford University HIV drug resistance database [37], were observed in up to 88% of RT gene sequences in 1996 (Figure 3A). The numbers of HIV-1 sequences containing drug-resistance mutations, gradually decreased after the introduction of cART in 1996, and the decline continued after that time probably due to the introduction of better tolerated drug regimens and increased patient adherence.

The number of key mutations in PR (Figure 3B) and RT (Figure 3C) was plotted. HIV-1 sequences with multiple key mutations were observed mainly from 1998-2007 for RT and from 2000-2007 for PR, roughly coinciding with the clinical introduction of these drugs. However, data from more recent years (>2009) indicate that sequences with more than 2 (PR) or 5 (RT) key mutations are observed less frequently, probably due to the development of optimized drug-regimens that improve therapy adherence and consequently plasma drug levels and antiviral activity. It thus seems that HIV-1 variants within the Amsterdam epidemic are not able to preserve these drug-related mutations, which may imply that there is a significant fitness cost involved and that compensating changes are rare [38]. We also described drug-dependent HIV-1 variants that will rapidly disappear in an epidemic upon transmission in the absence of the drug [39].

### A changing epidemic through import of novel HIV-1 variants

**Figure 4:**
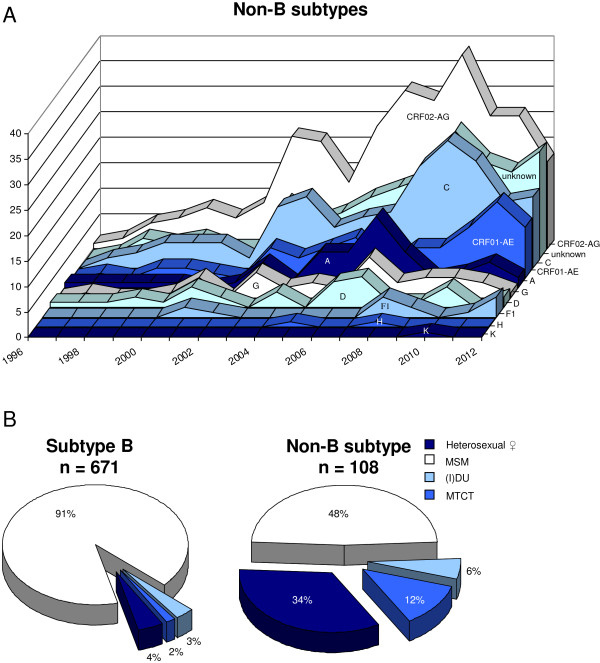
**HIV-1 subtype distribution in the AMC. A**. Numbers of HIV-1 non-B subtypes detected in patients visiting the AMC (Amsterdam, the Netherlands) from 1996-2012. “Unknown” consists of circulating recombinant forms (CRFs) other than CRF01_AE and CRF02_AG as well as difficult to type, complex mosaic strains and possible new subtypes. **B**. Percentages of HIV-1 B and non-B subtypes in the patient population according to the transmission risk group of HIV-1 that could be retrieved from the database. The total number of sequences (N) analysed in each group is indicated.

Subtype B has always been the most prevalent HIV-1 subtype that circulated in the Netherlands [40], most likely imported through drug users from the USA to initiate the epidemic [41]. However, since the late 1990s several non-B subtypes have been detected, usually linked to immigration from HIV-endemic areas [42]. Subtype B continues to dominate the epidemic, but substantial numbers of patients are infected with CRF01_AE, CRF02_AG and subtype C (Figure 4A). The latter two strains are mainly detected in heterosexual immigrants, while the former is present in MSM. Infections with untypable HIV-1 strains, including complex mosaic recombinant strains, are also on the rise from 2004 onwards with a steep ascend in 2008-2010 (Figure 4A). Subtype B strains are most prevalent in MSM, with very low percentages below 4% in other risk groups (Figure 4B). Of the non-B subtypes identified, 48% are carried by MSM, 34% by the heterosexual risk group, and 12% by children in the MTCT group (Figure 4B). HIV-1 subtypes differ in replication kinetics [43], e.g. basal transcription levels vary [44], as well in the induction of disease progression [45,46]. It is therefore important for patient monitoring to analyze changes in subtype distribution over time.

A remarkable case was identified in our diagnostic unit in 2000. An HIV-1 infected patient from the Netherlands yielded an unusual HIV-1 genotype that did not cluster with subtype B or any of the other subtypes [47]. The patient had worked as sailor from 1985 till 1989 and was probably infected via a heterosexual contact in Africa or perhaps Asia. We presented the full-length genome sequence of this exotic HIV-1 variant and determined that the genetic distance with known subtypes fell within the range of intersubtype and intersubsubtype distances. Thus, we could not conclude on this isolate being a novel subtype, a subsubtype or an outlier variant of the most closely related subtype K [47]. Similar sequences of other patients are needed to draw more firm conclusions and at least two epidemiologically unrelated patients with a similar HIV-1 variant are required to formally designate a new HIV-1 subtype. We also probed some biological properties of this virus, but did not reveal any particular assets. Because the patient was prescribed antiretroviral therapy, we were also able to monitor the appearance of drug-resistance mutations, which did not reveal major differences with the regular subtype B viruses.

Overall, our sample set has become more enriched for exotic HIV-1 variants, which is illustrated by the steep increase in the number of untypable sequences in the regular PR/RT genotyping performed. Likewise, we see a steady increase in the number of non-B subtypes in Amsterdam.

### A changing epidemic, but is the virus changing?

We described that the Dutch epidemic is changing with respect to the influx of new HIV-1 variants and subtypes. But is the virus that has spread in Amsterdam since 1983 also evolving? At the population level, one can monitor temporal trends in the pVL and CD4+ T-cell counts to reveal changes in the replication capacity and/or virulence of the respective HIV-1 strains. These values are important as they correlate with disease progression and the chance of HIV-1 transmission [12,48]. In Amsterdam, the set-point HIV-1 pVL of subtype B infected individuals was found to be higher in patients infected in more recent calendar years, with an accompanying decrease in CD4+ T-cell count [49,50]. Other studies have described similar trends [51-53], whereas others found no evidence for such temporal changes [54,55].

To address this experimentally, we have measured the in vitro replication capacity or fitness of early versus late Amsterdam HIV-1 isolates. Changes in virulence and fitness of viral pathogens in epidemics are common. For HIV-1, increases in viral fitness have been reported within patients, while bottlenecks occurring during transmission can reduce the fitness [56,57]. Early replication studies suggested that HIV-1 attenuates over time within an epidemic, probably due to repeated serial transmission bottlenecks [58]. However, one should carefully match the early and late samples for viral subtype and duration of the infection. Twenty-five early and late HIV-1 subtype B isolates, carefully matched for seroconversion time, were tested in replication and competition assays on peripheral blood mononuclear cells [59]. We observed a clear trend of increasing fitness over time in the HIV-1 epidemic of Amsterdam. This in vitro virus characteristic may constitute one of the determinants of clinical virulence, but this will be hard to assess in this era of antiretroviral therapy.

We have also focused on the molecular description of genetic defects in viruses of patients with a particular low pVL and slow disease progression. Such genetic markers that correlate with delayed disease progression have been identified in several HIV-1 proteins (see references in [60]). The Amsterdam patient under study had a low pVL of less than 1000 copies/ml for 2 years after seroconversion [60], which is observed in less than 1% of the individuals in the ACS cohort. This may suggest that the patient was infected with a poorly replicating virus. Indeed, a unique sequence insertion in the dimerization initiation site (DIS) of the untranslated 5’ region of the HIV-1 RNA genome was found that abolished RNA dimerization, one of the molecular hallmarks of retroviral replication [60]. Subsequent changes over time restored this important RNA function, in line with an increase in the pVL to 50.000 copies/ml. The pVL measured at set-point [61] may be influenced by host genetic factors [62,63], but can clearly also be influenced by viral genetic factors. The mutations that influence pVL at set-point can be hidden in every part of the HIV-1 genome. More dramatic mutations and deletions can be detected by sequencing and comparing complete genomes from other patients.

### Ongoing HIV-1 evolution, but a stable nucleotide count

Most virus genomes have a signature nucleotide composition, and HIV-1 is no exception, as its RNA genome is rich in A-nucleotides (approximately 35%) and poor in C-nucleotides (approximately 18%) [64-66]. Recently, we have described the extreme stability of this nucleotide composition over 30 years of evolution in the worldwide HIV-1 epidemic [67]. We plotted the nucleotide composition of PR and RT, which are sensitive to antiretroviral drug pressure, from patient samples sequenced in Amsterdam between 1995 and 2012 (Figure 3A). The percentages of all four nucleotides were found to be extremely stable in this genomic region over this period, and similar to older HIV-1 sequences. This suggests that during the Dutch epidemic HIV-1 is changing its virulence, but not its overall nucleotide composition. A recent study indicated that HIV-1 is able to replicate efficiently in cell culture after recoding parts of the gag or protease gene to reflect human codon usage [68]. These pilot results may suggest that changes in nucleotide composition do not always interfere with HIV replication capacity and can be tolerated. The major factor contributing to genome composition stability in HIV-1 remains to be elucidated.

### HIV-1 superinfections

The large number of recombinant strains in the HIV-1 epidemic suggested already in the early years that dual infections with different HIV-1 strains should occur. Dual infections were supposed to occur before seroconversion, because the first HIV-1 infection could protect against subsequent infections. It took until 2002 before the first HIV-1 superinfections (= infection with a different strain after seroconversion) were reported [69-71]. We reported the first triple HIV-1 infection in 2005 [72]. This subtype B infected patient was superinfected with a second subtype B strain and subsequently by a CRF01_AE strain. All three strains could be detected for several years in blood and seminal plasma, but the two superinfecting strains replicated to a higher level than the original virus [73].

We developed several methods to identify superinfections in clinical cohorts. The first method focused on patients with a sudden rise in pVL of at least 10-fold, which was found to predict superinfection in 2 of 14 patients [74]. Second, we described the use of degenerate base codes in the PR/RT genotyping sequence, which is a population sequence performed in routine clinical care. Multiple degenerate base code counts of ≥34 were found to be a strong indicator of superinfection [75].

We were interested to study the clinical outcome of dually infected patients. Several pieces of evidence, based on in vitro virus replication studies, infection of animal models and mathematical modelling, suggest that superinfection leads to increased virulence and a higher pVL [76]. Virus competition experiments with HIV-1 subtype B strains from two superinfected patients suggested that the superinfecting strain has a significant replicative advantage over the initial strain [77]. Follow-up in a small cohort of patients suggested that dual infection is significantly associated with increased CD4+ T-cell decline (p = 0.0001 in a multivariate analysis), and earlier start of antiretroviral therapy (p < 0.0001) [78].

The incidence of HIV-1 dual infections is increasing in Amsterdam from an absence in early years to 1.0-2.4% in 2003-2007 [79,80]. The clinically unfavourable outcome of dual infection should translate into preventive strategies, in particular because dual infection is associated with risk-taking behaviour that has been reported to increase in recent years [81].

### More sensitive HIV-1 detection

Although pVL is traditionally used as the biomarker of HIV-1 replication [82], the introduction of suppressive cART necessitated the development of alternative virological biomarkers for monitoring the therapy response, as pVL is undetectable by commercial assays in most of the cART-treated patients. In the ideal situation, such markers should be predictive of cART complications (e.g. therapy failure due to suboptimal adherence) and indicate the need for intervention, e.g. a change in drug-regimen. Quantitation of cell-associated (CA) HIV biomarkers provides an alternative to the measurements of pVL [83], but to be able to measure minute amounts of HIV nucleic acids in limited biological material from patients on suppressive cART, very sensitive assays are required.

We have developed sensitive and precise assays based on innovative semi-nested real-time PCR technology for quantitation of CA HIV unspliced (us) and multiply spliced RNA and total CA HIV DNA [84]. The addition of a limited-cycle preamplification step before the real-time PCR resulted in large improvements in both assay sensitivity and accuracy (especially in the lower quantitative range). This semi-nested real-time PCR is superior to the conventional single-step real-time PCR in the power of detection and quantitation of HIV-1 RNA and DNA [84]. Importantly, because we use only 15 cycles in the first PCR, preamplification does not interfere with the linearity of the standard curves throughout the whole range of serial dilutions (R^2^ > 0.99). The assay allows routine detection and quantitation of HIV RNA and DNA in the vast majority of PBMC samples of patients on cART with undetectable pVL [84-86]. Notably, this technology dramatically reduces the necessary amount of starting biologic material.

Using the semi-nested real-time PCR technology, we have shown that:

(i.) in untreated patients during the asymptomatic phase of HIV infection with steady-state plasma viremia, the levels of HIV-1 usRNA in PBMC increased significantly in time [87];

(ii.) in patients on suppressive cART, the levels of HIV- 1 usRNA in PBMC were predictive for therapy failure [85], an observation that was recently confirmed by an independent study that used a completely different assay for usRNA [88];

(iii.) in patients on ART with long-term virological success, modest nonadherence to ART caused significant longitudinal increase in the levels of usRNA in the absence of virological rebound in plasma [86];

(iv.) usRNA is a correlate of natural HIV control, as levels of usRNA in natural controllers were significantly lower than in patients on ART, while, importantly, T-cell proliferative responses to Gag and Pol peptides were higher [89].

These encouraging observations suggest that CA HIV-1 RNA is a significant and sensitive marker that allows monitoring the size and dynamics of the “active viral reservoir” in patients under cART. By contrast, total viral DNA is a marker of the “total viral reservoir”, which consists mostly of infected, non-producing cells. Moreover, our results show that CA HIV-1 RNA is a significantly more sensitive correlate of viral control than pVL, at least when the latter is measured by an assay with a detection limit of 40-50 copies/ml.

### HIV-diagnostics in resource-limited settings

The above described viral quantification method is a further example of the thriving merger between biomedical science and patient care in the Western world. However, assays like this are hardly applicable to resource-limited settings where most HIV-infected patients are living [90].To be useful in these settings, methods should be low cost, low complexity and preferably avoid cold-storage requirements. HIV rapid tests that employ saliva to detect antibodies against HIV have greatly simplified HIV screening and early diagnosis in developing countries [22,90]. However, now that ART availability is expanding in the developing world, affordable methods to monitor treatment efficacy and virological failure are needed to avoid the spread of drug-resistant HIV strains [91]. Although portable CD4+ T cell counters are on the market for use in resource-limited settings, the CD4+ T cell count is not the most optimal parameter to monitor treatment failure (see: [90]). Viral load measurements are superior to CD4+ T-cell counts, but as blood plasma requires cold storage, dried blood spot-based technologies would be the method of choice (see: [90,92]). For instance, a simple real-time PCR method using dried blood spots has been developed and tested by the Affordable Resistance Test for Africa (ARTA) program, in which the AMC participates [93]. Dried blood spots can also be analysed for drug-resistance mutations [94], preferably using simplified methods that can be used locally [95].

### A single case of seroreversion

As a retrovirus, HIV-1 induces a chronic infection that leads to a broad and well-sustained antibody response against multiple viral proteins. Seroconversion therefore occurs in all HIV-1 infected individuals, and antibody levels are maintained until the terminal AIDS phase in the majority of subjects. Seroreversion can be observed in non-infected children born to HIV-infected mothers that gradually loose their maternal antibodies [96,97] and also in exceptional AIDS patients with little remaining B-cell activity [98], but not in the bulk of HIV-1 infected patients [99].

However, we reported HIV-1 seroreversion in a patient that was treated with cART during acute infection and with a fully reactive Western blot [100]. Another seroreversion case was reported in 2005 that was also treated with cART during primary infection. In this case, an incomplete HIV-1 antibody evolution pattern was revealed, suggesting that treatment was started before the antibody repertoire was completed [101]. Larger studies involving adult and young patients treated early in the infection suggested that HIV-1 seroreversion is not uncommon [102,103]. In contrast, seroreversion is rare in patients treated with cART later in infection, even when long-term undetectable pVL is achieved. One study reported no seroreversions in 80 patients tested [104] and another study found 1 out of 84 patients to have seroreverted [105].

## Conclusions

The HIV-1 epidemic has triggered an evolution in patient care with the implementation of molecular biology techniques, which over 30 years have been optimized considerably. The careful collection of thousands of patient samples for these clinical tests has benefitted basic scientific research enormously, as illustrated for the AMC hospital in this review. Among the 50992 patients analyzed, we described unique HIV-1 variants in individual patients, including exotic mutants and unfamiliar strains. We performed studies to probe virus evolution, showing a rapidly changing pathogen (virulence, drug resistance, complex mosaic strains or undetermined subtypes), but at the same time very constant properties (genomic nucleotide composition). In return, molecular virology research allowed the development of novel diagnostics, including the ultra-sensitive detection of HIV-1 RNA in cells instead of plasma.

## Competing interests

The authors declare that they have no competing interests.

## Authors’ contributions

BB conceived the review topic. BB, ACvdK and AOP drafted the manuscript. MB analyzed the data from the HIV databases, and generated the figures. MC, SJ and NKTB helped drafting the manuscript. All authors read and approved the final manuscript.
